# Registration of Vehicle-Borne Point Clouds and Panoramic Images Based on Sensor Constellations

**DOI:** 10.3390/s17040837

**Published:** 2017-04-11

**Authors:** Lianbi Yao, Hangbin Wu, Yayun Li, Bin Meng, Jinfei Qian, Chun Liu, Hongchao Fan

**Affiliations:** 1 College of Surveying and Geoinformatics, Tongji University, Shanghai 200092, China; lianbi@tongji.edu.cn (L.Y.); 90yayun_li@tongji.edu.cn (Y.L.); mengbin03@163.com (B.M.); 1qianjf@tongji.edu.cn (J.Q.); liuchun@tongji.edu.cn (C.L.); 2 Institute of Geography, Heidelberg University, Heidelberg D-69120, Germany; hongchao.fan@uni-heidelberg.de

**Keywords:** mobile mapping system, point clouds, panoramic image, registration, feature point, sensor constellation

## Abstract

A mobile mapping system (MMS) is usually utilized to collect environmental data on and around urban roads. Laser scanners and panoramic cameras are the main sensors of an MMS. This paper presents a new method for the registration of the point clouds and panoramic images based on sensor constellation. After the sensor constellation was analyzed, a feature point, the intersection of the connecting line between the global positioning system (GPS) antenna and the panoramic camera with a horizontal plane, was utilized to separate the point clouds into blocks. The blocks for the central and sideward laser scanners were extracted with the segmentation feature points. Then, the point clouds located in the blocks were separated from the original point clouds. Each point in the blocks was used to find the accurate corresponding pixel in the relative panoramic images via a collinear function, and the position and orientation relationship amongst different sensors. A search strategy is proposed for the correspondence of laser scanners and lenses of panoramic cameras to reduce calculation complexity and improve efficiency. Four cases of different urban road types were selected to verify the efficiency and accuracy of the proposed method. Results indicate that most of the point clouds (with an average of 99.7%) were successfully registered with the panoramic images with great efficiency. Geometric evaluation results indicate that horizontal accuracy was approximately 0.10–0.20 m, and vertical accuracy was approximately 0.01–0.02 m for all cases. Finally, the main factors that affect registration accuracy, including time synchronization amongst different sensors, system positioning and vehicle speed, are discussed.

## 1. Introduction

### 1.1. Background

The Mobile Mapping System (MMS) is an advanced system used to collect the environmental geospatial and texture data and consists of three main parts: mapping sensors, a positioning and navigation unit for spatial referencing, and a time referencing unit [1]. Since the first MMS was created by the Center for Mapping at Ohio State University [2,3], MMS has developed rapidly and become an important data source of 3D city modeling, indoor mapping and urban mapping and planning [1,4,5]. Other advanced MMSs have been developed and are used for real-time air pollution monitoring and health risk management [6,7].

Vehicle-borne MMSs are usually installed with a laser scanner and digital camera or video as mapping sensors [1]. Therefore, the integration of the data collected by different sensors, especially the fusion of point clouds and digital imagery, including depth imagery, has become an important research topic [8,9,10,11]. Panoramic cameras have been commonly utilized to replace the traditional digital camera in collecting texture information [12,13,14]. Compared with traditional digital cameras, panoramic cameras collect environmental data with a better field of view and accurate positioning module. Therefore, the fusion of panoramic images and other data from sensors, especially the point clouds from a laser scanner, is an important issue.

The following subsection presents several previous studies on the fusion of a point clouds and images, including panoramic images.

### 1.2. Previous Studies

Previous studies relating to the registration of panoramic images and the LiDAR (Light Detection And Ranging) point clouds describe four methods. The first method is a non-rigid ICP (Iterative Closest Point) algorithm proposed by [15], which incorporates a bundle adjustment for ICP processing and conducts a refinement using SIFT (Scale-Invariant Feature Transform) features detected from both kinds of datasets. The second method is a sensor-alignment based method, which extracts each CCD (Charge-Coupled Device) camera from the panoramic imaging system and obtains the accurate internal orientation parameters through calibration [16]. Third, a complex model from a world coordinate system to single CCD images was established to find the precise relationship between point and pixel. A collinear principle, which establishes the relationship between the center of the panoramic camera, the image point on the sphere, as well as the object point, was proposed by [17]. This method also uses the accurate relationship between GPS, POS (Positioning and Orientation System), and LiDAR sensors. The final method, the feature-line based registration model proposed by [18], extracts the linear features of the terrain from both LiDAR data and the panoramic images to establish the transformation model. All these methods were successfully applied to the registration of panoramic images and TLS (Terrestrial Laser Scanning) or MLS (Mobile Laser Scanning) point clouds. As the panoramic camera is always working during data collection, this will lead to situations where the registration between panoramic images with point clouds is a 1:N problem, as a point of the point clouds will find more than one pixel during registration.

Although only a few studies have addressed the registration between point clouds and panoramic images, the methods for the registration of point clouds and images can also be used if the panoramic images are separated into individual ones. These methods can be divided into two types.

The first type of registration method is based on the point clouds generated by laser scanners and digital cameras. An important parameter to describe this method is the overlapping rate of the images acquired by digital cameras. When the overlapping rate of digital images exceeds a set threshold, dense registration [19] or structure from motion (SFM) [20] algorithms can be applied to calculate the point clouds pixel by pixel for adjacent or unorganized images. Registration of the point cloud and digital imagery is transformed to registration between the point clouds from a laser scanner and point clouds from digital imageries by SFM or other methods. Several traditional or recently developed methods can be used in this situation, of which iterative closest point (ICP) [21] is the most common method. Several extensions and additional conditions based on the basic ICP algorithm, such as random sample consensus [22] and the least median of squares (LMS) estimators [23], have been developed to increase robustness and convergence and improve computational efficiency and performance. Various other methods based on the features extracted from both sources of the point clouds, such as corner points of buildings [24,25], polyline and polygon features [26,27,28], normal vectors of polygon features [29,30] and urban road networks [31], can also be applied. An important feature of this registration approach is that the acquisition view of the point clouds is different for the two technologies. For example, a terrestrial laser scanner acquires point clouds data on a terrain, and an unmanned aerial vehicle (UAV) acquires images from a low-altitude space. Good complementarity exists between the point clouds collected through these two methods, therefore, this registration method is commonly used to integrate point clouds, especially from complex buildings or other such infrastructure.

The second type of method is based on the features extracted from a point clouds and images. Before extracting and applying features for registration, the accurate relationship amongst sensors, such as digital cameras, GPS and IMU, should be calibrated and computed. Another pre-processing procedure for digital cameras is the calibration of the different lenses of cameras. Shift and rotation relationship data are important in the registration of different sensors. The authors of [32] calibrated the fixed mathematical relationship between laser scanners and digital cameras and synchronously acquired point clouds and image data to complete the registration. POS data is usually used to integrate a 3D point clouds and a 360° linear array panoramic camera [33]. The authors of [34] reviewed relevant feature-based methods for direct registration of point clouds and digital images. Current studies indicate that, compared with natural targets, man-made features are used more frequently [35]. Several easy-to-find, man-made features, such as linear edges [36,37], connected line segments [38], and planar features [39], are often used for registration. Several other invisible features, such as vanishing point [40] and mutual information [41,42], are also used. Amongst these methods, visible and linear feature extraction algorithms, including scale-invariant feature transform, are consistently employed [43,44]. UAVs have also been utilized to capture aerial images, and the registration of aerial-based point clouds and images has been studied [45].

### 1.3. Present Work

Several registration and data integration methods have been developed for the fusion of point clouds and digital images. However, from our perspective, these methods cannot be directly used to combine point clouds and panoramic images. For example, given that the overlap rate amongst the adjacent lenses of a panoramic camera is small, the dense registration method for point clouds generated by the photogrammetry method can only achieve a few point clouds. In addition, feature-based methods are usually developed to register point clouds and traditional digital images, and the application of these methods to different situations, such as panoramic images, requires further verification. Furthermore, as more than four lenses exist in a panoramic camera, the selection of corresponding imagery for a certain point is vital for registration. Therefore, a new method to register point clouds and panoramic images is required.

To address this issue, a new registration approach based on sensor constellation was developed in this study. This approach makes full use of the GPS and the panoramic camera’s position and orientation relationship. A segmentation feature point was computed based on the real-time sensor constellation. Using such segmentation feature points, point clouds acquired through different laser scanners can be divided into small blocks. Finally, points of each block were introduced to the geometric conversion model to compute the corresponding pixels in panoramic images. After that, the color or texture information of the panoramic images was extracted and fused with the 3D point clouds. Compared with other methods, considering the position of sensors was fixed after installation, the proposed method is simple, and suitable for large volume calculation.

## 2. Sensor Constellation of MMS

The sensor constellation of an MMS differs according to its application purpose. In this study, an MMS was designed to collect data from urban roads and extract the visible features, including symbols and markers for transportation and terrain objects around the roads. This MMS contained a panoramic camera, sectional scanners, GPS, IMU and other necessary sensors.

**Figure 1:**
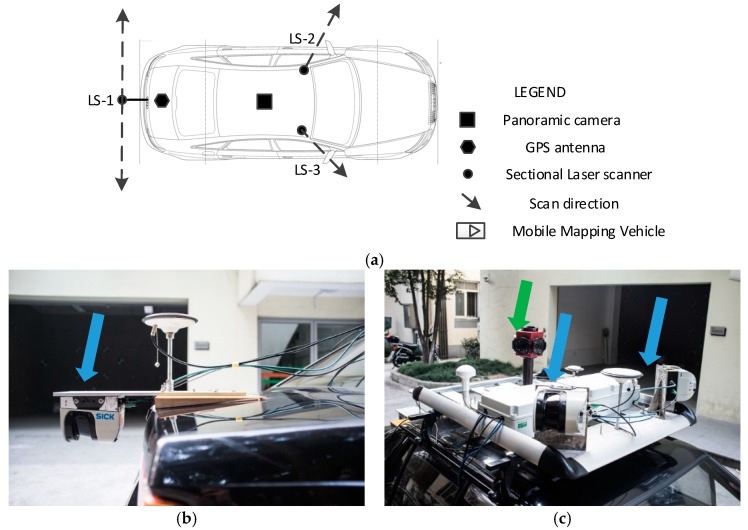
Main sensors installed in the mobile mapping system. (**a**) Spatial distribution of sensors; (**b**) rear-view laser scanner and GPS antenna; and (**c**) two side-view laser scanners and panoramic camera.

Three laser scanners were installed in different locations. Figure 1 shows the relationship between the panoramic camera, sectional laser scanners and GPS antenna. The first sectional laser scanner, see LS-1 in Figure 1, was installed in the rear of the vehicle to collect data from the top view. The other two laser scanners were installed on either sides of the roof of the vehicle to collect data on both sides of the road. LS-2 collected data from the left-front direction, and LS-3 acquired data from the right-front direction. Both LS-2 and LS-3 collected point clouds in a vertical plane. As the orientation and position relationship was accurately calibrated, the point clouds captured by these three laser scanners was treated with the same coordinate system for subsequent registration.

The panoramic camera used in this study was a LadyBug^®^ (FLIR Integrated Imaging Solutions, Inc., Richmond, BC, Canada), and was installed in the center of the vehicle roof (see Figure 1a and the green arrow in Figure 1c). The camera contained six high-definition lenses and captured images from six directions simultaneously. The sampling rate was 1.0 Hz. NMEA (National Marine Electronics Association) GPS signals were input into the panoramic camera in real-time to record the position of the panoramic camera.

The sectional laser scanners were SICK^®^ LMS 511 Pro. (SICK Vertriebs-GmbH, Düsseldorf, NRW, Germany). The maximum scanning distance of this kind of laser scanner was 80 m, and the field of view reached 190°. The scanning frequency was 100 Hz, and the angle resolution was 0.166°. The main original data collected by the laser scanner were time, distance, intensity, and scan angle.

**Figure 2:**
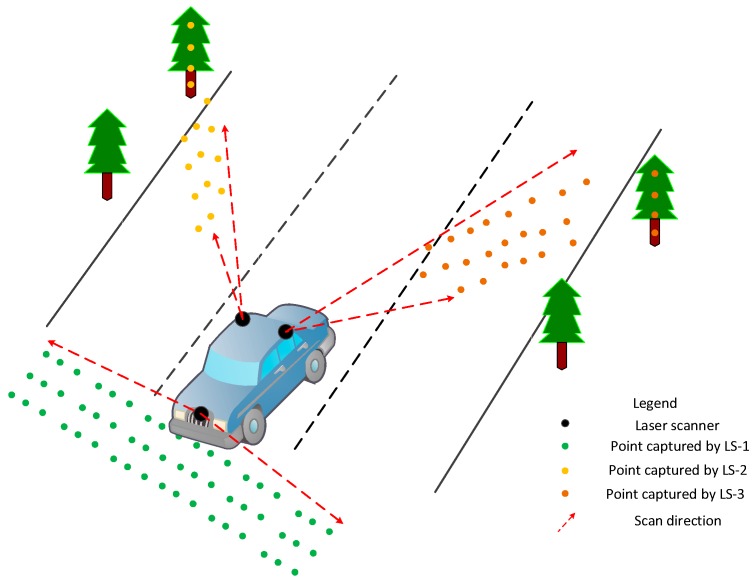
Point cloud distribution of the proposed MMS.

According to the data-processing model, an integration system was developed to collect sensor data and compute point clouds data from the original IMU, GPS and laser scanner so that a 3D point clouds of surrounding objects could be obtained. The MMS comprised of three sectional laser scanners where two were installed in the front part of the vehicle, and the other one was installed in the rear. The point clouds from different scanners were separated from the original point clouds. Figure 2 shows the spatial distribution of the point clouds captured by the different laser scanners. The green points were captured by LS-1 and are located in the road and go along with the movement of the vehicle. The other points in yellow or brown were located on the roadside and were captured by LS-2 and LS-3. The scan planes of LS-2 and LS-3 were perpendicular to the horizontal plane.

## 3. Registration of Mobile Point Clouds and Panoramic Images Based on Sensor Constellations

In this section, the registration procedures of the mobile point clouds and the panoramic images are introduced based on the sensor constellation of the MMS. Section 3.1 outlines the flowchart of the proposed method and Section 3.2, Section 3.3 and Section 3.4 introduce the detailed processing steps.

### 3.1. Flowchart of Proposed Method

**Figure 3:**
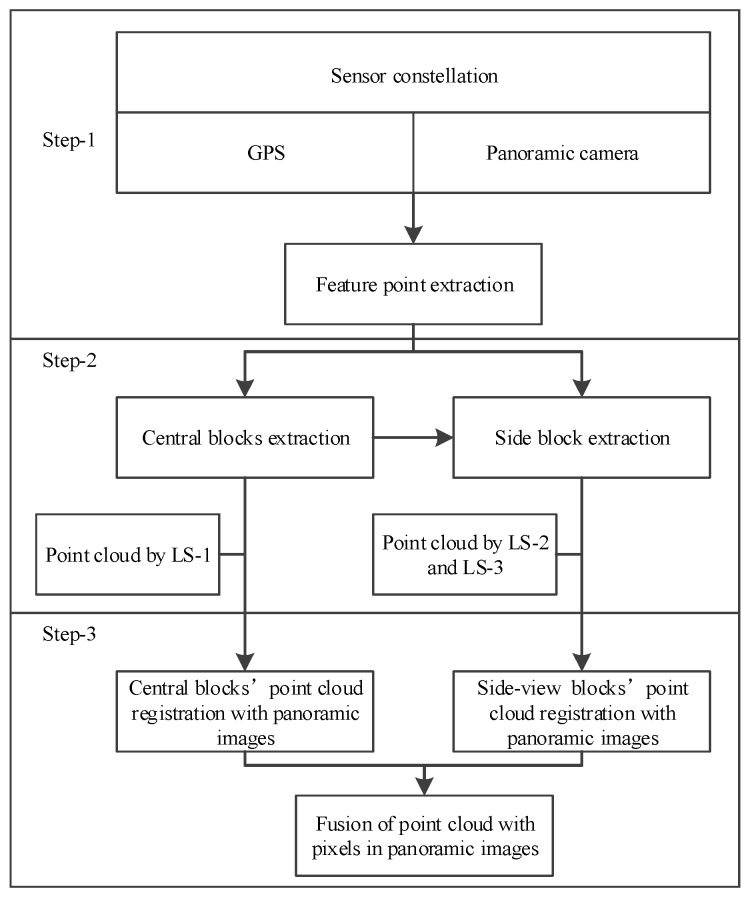
Flowchart of point cloud division and block extraction.

Three main steps were included in the sensor-constellation based method (Figure 3). First, in order to separate the whole road’s point clouds into small blocks, the sensor constellation (mainly the panoramic camera and the GPS) were analyzed and a segmentation feature point extraction model was proposed. As both the panoramic camera and GPS contained a positioning module, the position of the panoramic camera and the GPS could always be obtained. This ensured that the feature point could always be acquired while MMS was travelling. This step will be explained in Section 3.2 in detail.

After the segmentation feature point was obtained, a polygon area was extracted and defined as the central block for LS-1 (discussed in Section 3.3.1). The corner points of the central block were also computed, and the side blocks were fixed (discussed in Section 3.3.2). The central and side blocks were used to segment the whole point clouds into small blocks.

Finally, each point in the small blocks was selected to find the corresponding pixel in the panoramic image using the sensors’ relationship matrixes introduced in Section 3.4. An image search strategy is also provided in this subsection.

### 3.2. Segmentation Feature Point Extraction Based on Sensor Constellation

**Figure 4:**
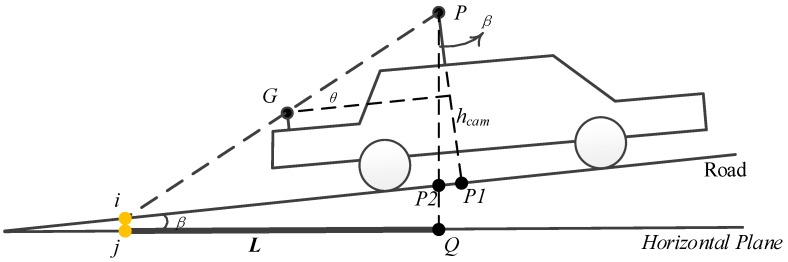
Principle of segmentation point calculation.

In this subsection, a feature point was extracted according to the sensor constellation between the GPS and panoramic camera. As the position of the GPS and panoramic camera can always be obtained, the relationship between the two sensors, as well as the vehicle and the ground is easy to rebuild. A diagram of the principle of segmentation feature point extraction is shown in Figure 4.

$G\left(x_{gps}^{T},y_{gps}^{T},h_{gps}^{T}\right)$ is assumed to be the coordinate of GPS antenna at a certain time (T), and $P\left(x_{cam}^{T},y_{cam}^{T},h_{cam}^{T}\right)$ is the coordinate of a panoramic camera at the same time. These positions can be obtained according to GPS observation and the relationship between the GPS and the panoramic camera.

Let β be the road inclination angle at a certain position, which can be obtained by GPS according to the adjacent epochs or the point clouds captured nearby. In this study, previous epochs of GPS were utilized to calculate the inclination angle.

$h_{cam}$ is the distance from the centre of the panoramic camera to the road (see line P-P1 in Figure 4) and will stay the same after the panoramic camera has been installed. The horizontal plane is the projection plane with a certain elevation h and is also the computing side.

*i* is the intersection point between line *PG* and the road. *L* is the line between point *j* and *Q*, which is the projection point of *i* and *P* to the horizontal plane. In this study, point *j* was regarded as the segmentation feature point.

Therefore, the vertical angle, θ, can be obtained with

(1) $$\theta +\beta =arcsin\left(\frac{z_{cam}^{T}-z_{gps}^{T}}{\sqrt{{\left(x_{cam}^{T}-x_{gps}^{T}\right)}^{2}+{\left(y_{cam}^{T}-y_{gps}^{T}\right)}^{2}+{\left(z_{cam}^{T}-z_{gps}^{T}\right)}^{2}}}\right).$$

The distance of line *i-P*2, $d_{i-P2}$, can be obtained with

(2) $$d_{i-P2}=d_{i-P1}-d_{P1-P2}=\frac{h_{cam}}{\tan\theta }-h_{cam}\times \tan\beta .$$

Given that *L* is the projection of line *i − P*, which is also the projection of line *i − P*2, it can be calculated with

(3) $$L=d_{j-Q}=d_{i-P2}\times \cos\beta =\left(\frac{h_{cam}}{\tan\theta }-h_{cam}\times \tan\beta \right)\times \cos\beta .$$

**Figure 5:**
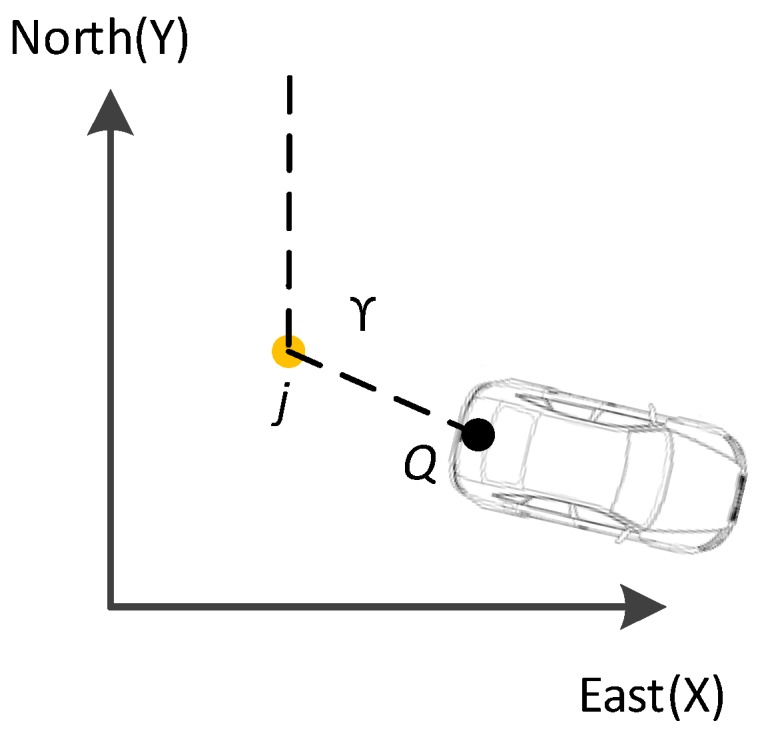
Relationship of the segmentation feature point and mobile mapping vehicle.

Figure 5 shows the relationship of the segmentation feature point (*j*) and mobile mapping vehicle. 

The coordinate of segmentation feature point $j\left(x_{j}^{T},y_{j}^{T},h_{j}^{T}\right)$ can be calculated according to azimuth angle γ and projected panoramic camera coordinate $Q\left(x_{cam}^{T},y_{cam}^{T},h\right)$ at a certain time (*T*) as follows:

(4) $$\{\begin{array}{c} x_{j}^{T}=x_{cam}^{T}-L\times \cos\gamma =x_{cam}^{T}-\left(\frac{h_{cam}}{\tan\theta }-h_{cam}\times \tan\beta \right)\cos\beta \cos\gamma \\ y_{j}^{T}=y_{cam}^{T}-L\times \sin\gamma =y_{cam}^{T}-\left(\frac{h_{cam}}{\tan\theta }-h_{cam}\times \tan\beta \right)\cos\beta \sin\gamma \\ h_{j}^{T}=h \end{array}$$

### 3.3. Division of Original Points into Blocks

#### 3.3.1. Division of the Point Clouds Captured by LS-1

After the location of the segmentation feature point was calculated, the road’s point clouds was divided to separate the large volume of points into small blocks.

**Figure 6:**
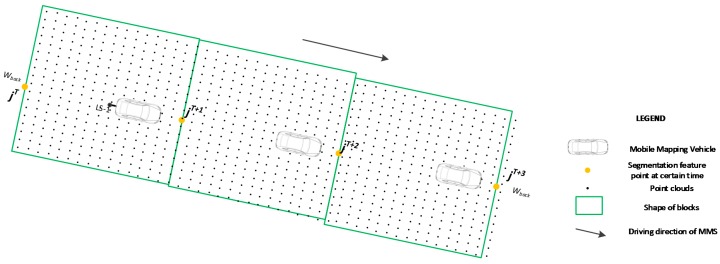
Block division for the point clouds captured by LS-1.

Based on the data collection frequency of the panoramic camera, each segmentation point during the time the panoramic camera was operational was computed. A threshold, $W_{back}$, which indicates the width of the block shape, was used to determine the width of the polygon (Figure 6), and the length of the block shape was calculated by the adjacent segmentation feature points (*j^T^* and *j^T+^*^1^). Next, the block shape was fixed (see green box in Figure 6) and the point clouds contained in the polygon was stored as a small block for further processing.

Considering that the field angle of the sectional laser scanner used in this study was more than 180°, the distance of the scan line is extremely large in theory. However, the threshold $W_{back}$ limits the area to the most central part of the scan lines as a point’s accuracy in this area is better than that of other points. Furthermore, the points located at the side were overlapped by the point clouds from the other laser scanner. Therefore, the points that were out of the block shape were not used in the registration technique described in Section 4.

#### 3.3.2. Division of the Point Clouds Captured by LS-2 and LS-3

The block shape for LS-1 can be accurately determined by the segmentation points and the parameter $W_{back}$; however, the method introduced in Section 3.3.1 cannot be used for the two other laser scanners (LS-2 and LS-3) as no obvious target exist at the side of the vehicle. Therefore, a two-step method was introduced to divide the point clouds captured by LS-2 and LS-3 into small blocks.

**Figure 7:**
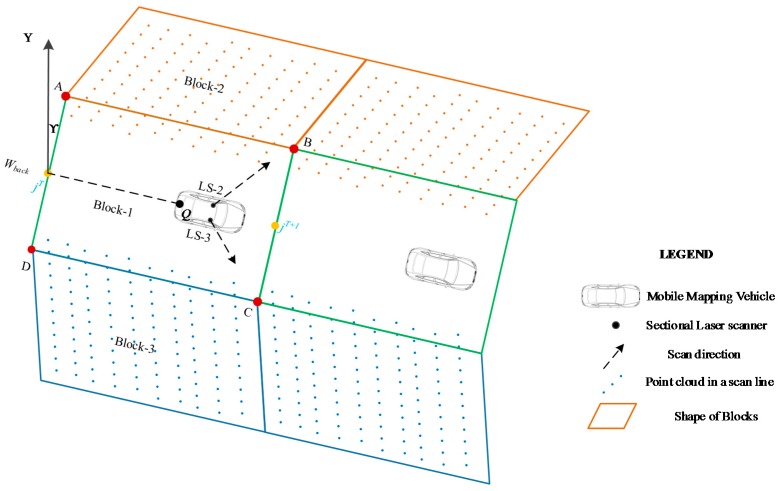
Block division for the point clouds captured by LS-2 and LS-3.

First, the coordinates of four corner points were obtained at each time point (see A, B, C and D in Figure 7). Given that the coordinates of the feature points, $j^{T}\left(x_{j}^{T},y_{j}^{T},h\right)$ and $j^{T+1}\left(x_{j}^{T+1},y_{j}^{T+1},h\right)$, were obtained with Equation (4), the corner points could also be calculated. For example, the coordinate of point $A\left(x_{A}^{T},y_{A}^{T},h_{A}^{T}\right)$ at time T can be calculated as

(5) $$\{\begin{array}{c} x_{A}^{T}=x_{j}^{T}+\frac{W_{back}}{2}\times \sin\left(\gamma -90{}^{\circ}\right)\\ y_{A}^{T}=y_{j}^{T}+\frac{W_{back}}{2}\times \cos\left(\gamma -90{}^{\circ}\right)\\ h_{A}^{T}=h \end{array}$$

where γ is the azimuth angle of line *jQ* (Figure 5).

Second, the shape of the block was determined as per the corner points and the scan direction of the laser scanner (LS-2 and LS-3). The scan direction of the laser scanner was determined when the sensor was installed.

Similar to the point clouds captured by LS-1, most of the point clouds captured by LS-2 or LS-3 were inserted into blocks for registration. Few points were removed as they were overlapped by the points of the central block captured by LS-1. The blocks from a different laser scanner were adjacent, albeit without overlap.

### 3.4. Registration of a Block’s Point Clouds and Panoramic Images

The point clouds of MMS are continuously collected and represent the locations of surrounding objects. The frequency of the laser scanner is higher than that of the panoramic camera, so the point clouds are continuous while panoramic images are discontinuous. After processing via the methods introduced in Section 3.2 and Section 3.3, the point clouds were divided into small blocks. The points in each block were used to register the images collected by the panoramic camera.

As the purpose of the registration between the point clouds and panoramic images was to fuse the texture information of the images with the geometric information of the point clouds, each point extracted from the blocks was selected and converted into the pixel coordinate of the panoramic camera via the local coordinate system of the MMS and the local coordinate system of the panoramic camera. If a proper pixel was found in the relevant lens’ imagery, the color information was stored. Otherwise, if no proper pixel was found, then the point was removed. As these steps are quite simple using photogrammetry principles and equations in textbooks, this part of the processing is omitted in this paper.

Before applying the point cloud coordinate conversion, some pre-processing, such as distortion correction for the panoramic camera, interior orientation element calibration for each lens of the panoramic camera and geometric relationship calibration between the local coordinate system of the panoramic camera and MMS, needs to be conducted. In this study, several traditional methods including [46,47,48,49] were used.

$\left(X_{k}^{w},Y_{k}^{w},Z_{k}^{w}\right)$ is assumed to be the coordinate of point (*k*) in the world coordinate system, $\left(X_{k}^{pan},Y_{k}^{pan},Z_{k}^{pan}\right)$ denotes the coordinate of point (*k*) in the panoramic camera coordinate system and $\left(X_{k}^{mms},Y_{k}^{mms},Z_{k}^{mms}\right)$ denotes the corresponding coordinate of point (*k*) in the MMS coordinate system. According to the point cloud calculation method, the relationship between $\left(X_{k}^{w},Y_{k}^{w},Z_{k}^{w}\right)$ and $\left(X_{k}^{mms},Y_{k}^{mms},Z_{k}^{mms}\right)$ is

(6) $$\left(\begin{array}{c} X_{k}^{w}\\ Y_{k}^{w}\\ Z_{k}^{w} \end{array}\right)=\left(\begin{array}{c} \Delta X_{mms}^{w}\\ \Delta Y_{mms}^{w}\\ \Delta Z_{mms}^{w} \end{array}\right)+R_{mms}^{w}\times \left(\begin{array}{c} X_{k}^{mms}\\ Y_{k}^{mms}\\ Z_{k}^{mms} \end{array}\right).$$

Therefore,

(7) $$\left(\begin{array}{c} X_{k}^{mms}\\ Y_{k}^{mms}\\ Z_{k}^{mms} \end{array}\right)={\left(R_{mms}^{w}\right)}^{-1}\left(\begin{array}{c} X_{k}^{w}-\Delta X_{mms}^{w}\\ Y_{k}^{w}-\Delta Y_{mms}^{w}\\ Z_{k}^{w}-\Delta Y_{mms}^{w} \end{array}\right)$$

where $R_{mms}^{w}$ and ${\left(\Delta X,\Delta Y,\Delta Z\right)}_{mms}^{w}$ are the transformation parameters from the MMS coordinate system to the world coordinate system, which can be achieved during point cloud computation processing. The coordinate of point *k* in the MMS coordinate can be achieved using Equation (7).

Next, $\left(X_{k}^{mms},Y_{k}^{mms},Z_{k}^{mms}\right)$ was transferred to the coordinate system of the panoramic camera via

(8) $$\left(\begin{array}{c} X_{k}^{pan}\\ Y_{k}^{pan}\\ Z_{k}^{pan} \end{array}\right)=\left(\begin{array}{c} \Delta X_{mms}^{pan}\\ \Delta Y_{mms}^{pan}\\ \Delta Z_{mms}^{pan} \end{array}\right)+R_{mms}^{pan}\times \left(\begin{array}{c} X_{k}^{mms}\\ Y_{k}^{mms}\\ Z_{k}^{mms} \end{array}\right)$$

where $\left(\Delta X_{mms}^{pan},\Delta Y_{mms}^{pan},\Delta Z_{mms}^{pan}\right)$ and $R_{mms}^{pan}$ were determined after the panoramic camera was installed and can be obtained via the calibration of the MMS system. Finally, in the panoramic camera coordinate system, the pixel coordinate (*x, y*) of an image can be computed according to the following collinear equation.

(9) $$\{\begin{array}{c} x=-f\frac{a_{1}\left(X_{k}^{pan}-X_{s}\right)+b_{1}\left(Y_{k}^{pan}-Y_{s}\right)+c_{1}\left(Z_{k}^{pan}-Z_{s}\right)}{a_{3}\left(X_{k}^{pan}-X_{s}\right)+b_{3}\left(Y_{k}^{pan}-Y_{s}\right)+c_{3}\left(Z_{k}^{pan}-Z_{s}\right)}\\ y=-f\frac{a_{2}\left(X_{k}^{pan}-X_{s}\right)+b_{2}\left(Y_{k}^{pan}-Y_{s}\right)+c_{2}\left(Z_{k}^{pan}-Z_{s}\right)}{a_{3}\left(X_{k}^{pan}-X_{s}\right)+b_{3}\left(Y_{k}^{pan}-Y_{s}\right)+c_{3}\left(Z_{k}^{pan}-Z_{s}\right)} \end{array}$$

where ($X_{s},Y_{s},Z_{s}$) refer to the linear elements of the exterior orientation of the panoramic camera. These parameters can be obtained as per the GPS and IMU observations, as well as the installation relationship of the panoramic camera and GPS. $\left(a_{1},b_{1},c_{1},a_{2},b_{2},c_{2},a_{3},b_{3},c_{3}\right)$ are the elements of the direction cosine matrix, which can also be fixed at a certain time.

Considering that the panoramic camera used in this study contained six different lenses, for a normal registration process, the corresponding pixel should be searched for each image captured by each lens. However, this procedure reduces calculation efficiency so a registration strategy was adopted in this study.

**Figure 8:**
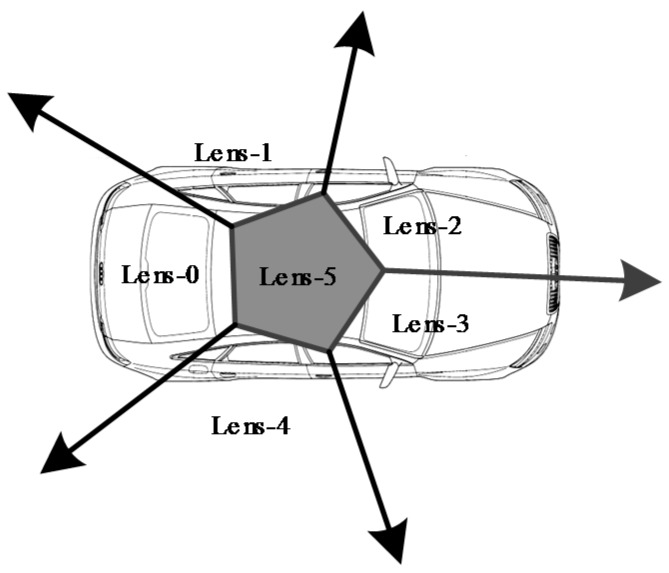
Spatial distribution of field of view of the different lenses of the panoramic camera.

Figure 8 shows the observation field of view of each lens of the panoramic camera. According to the installation relationship between the panoramic camera and laser scanner, the field of view of a lens is always associated with a certain laser scanner. Therefore, we performed search processing according to improve search efficiency (Table 1).

Using the coordinates of the point and the parameters listed in Equations (6)–(9), the corresponding pixel can be directly found and there is an obvious difference when registering the different laser scanners. However, when applying Equation (9), the lens’ direction cosine matrix should be given as per Table 1.

## 4. Case Studies

### 4.1. Case Area

Four areas in Shanghai were selected to validate the proposed method. Given that the main purpose of the MMS was to obtain the symbols and markers as well as the relevant objects of roads, these test cases corresponded to four different types of roads: overpass, freeway, tunnel, and surface roads with intersections. The main information of these test cases and the data collection environments and parameters are listed in Table 2.

### 4.2. Registration Results

#### 4.2.1. Efficiency Evaluation for Different Laser Scanner’s Point Clouds

To evaluate the efficiency of the registration between different laser scanners and the different lenses of a panoramic camera, the total points, valid matched points, match rate, and computation time were summarized after registration processing. During the processing, the $W_{back}$ was 160.0 m, which was twice the maximum distance of the laser scanner. Therefore, most of the point clouds was divided into small blocks and used for registration. The total points captured by different laser scanner, matched points as well as the match rate and computation time are shown in Table 3.

From the information presented in Table 3, we see that the number of points of LS-1 is higher than LS-2 and LS-3 as LS-1 was designed to collect points of the road and most of the laser beam was reflected. However, the other two laser scanners were designed to collect points around the road, and some of the laser beam was missing, as no targets exist in the air.

As seen in Table 3, most of the points were successfully matched (with an average of 99.7%) with the corresponding pixels in the panoramic images. Very few points were unmatched during the registration procedures. After summarizing the total points and computation time for each laser scanner, the average computation efficiency was 38,155 points/s, 23,870 points/s and 24,006 points/s for LS-1, LS-2 and LS-3, respectively (Table 4). The efficiency of LS-1 was 1.59 times higher than other two laser scanners, as the image search range was different (shown in Section 3.4).

#### 4.2.2. Visualization of Registration Results

**Figure 9:**
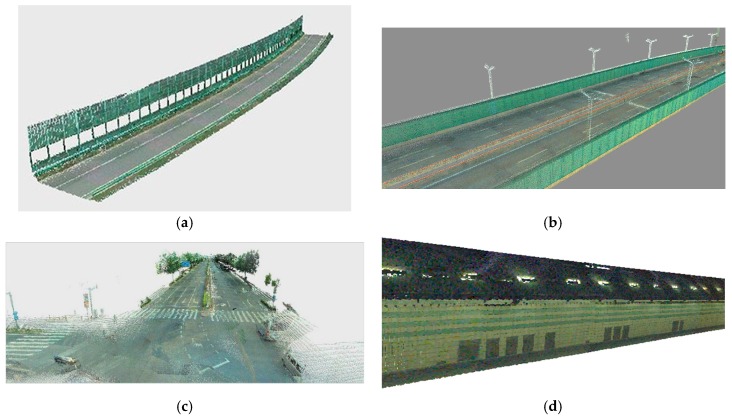
Visualization of four different types of roads. (**a**) Overpass; (**b**) freeway; (**c**) surface roads; and (**d**) tunnel.

To evaluate the registration results for different objects around the road completed by our proposed method, Figure 9 shows the visualization of four different types of roads. As the light conditions within the tunnel environment was poorer than the other roads, the brightness of the tunnel point clouds (see Figure 9d) after matching was lower than the other three examples. The main objects and the symbols around the roads—for example, solid/dash line, lights, central isolation belt, acoustic panels, crosswalk, left turn lane, trees, and some vehicles—could be accurately identified using the fused point clouds. This also enhanced the possibility of automatically extracting the objects and symbols around the roads.

### 4.3. Accuracy Evaluation

#### 4.3.1. Evaluation Method

**Figure 10:**
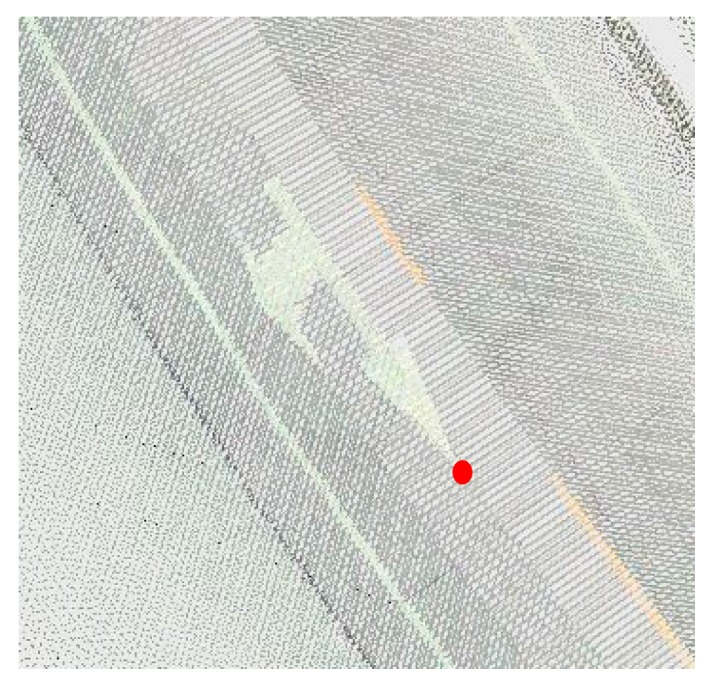
Selection of feature point for accuracy evaluation.

The distance of checkpoints before and after registration was used to evaluate registration accuracy. Given that the main purpose of this study was to fuse the texture information of an image with the geometric information of the point clouds, the feature points were manually selected based on the fused point clouds. For example, as the arrow can be manually found in both the original (rendered by intensity) and fused point clouds (rendered by color), the arrow in the road was then selected, and the top point of the arrow was used for comparison (Figure 10).

Supposing that the coordinate of the arrow point in the fused point clouds is $\left(X^{f},Y^{f},Z^{f}\right)$ and in the original point clouds is $\left(X^{o},Y^{o},Z^{o}\right)$, three indices can be obtained to evaluate the geometric accuracy of registration. $d_{H}$ indicates the horizontal offset for the arrowhead after registration.

(10) $$d_{H}=\sqrt{{\left(X^{f}-X^{o}\right)}^{2}+{\left(Y^{f}-Y^{o}\right)}^{2}}$$

(11) $$d_{V}=\left|Z^{f}-Z^{o}\right|$$

(12) $$d=\sqrt{d_{H}{}^{2}+d_{V}{}^{2}}.$$

#### 4.3.2. Evaluation Result

Twenty checkpoint pairs for each different road type were manually selected, and the relative indicators (described in Section 4.3.1) were computed to evaluate accuracy. The coordinates and the differences of checkpoints of each case are listed in Appendix A. The statistical results are shown in Table 5. $d_{H}$ indicates the horizontal offset, while $d_{V}$ indicates the horizontal offset. *d* is the total offset between before and after registration.

Table 5 indicates that plane geometric accuracy was approximately 0.10–0.20 m. Accuracy varied across the different environments, which may have been caused by several factors and will be analyzed in Section 4.4.

Although the tunnel elevation varied from −4.4 m to −40.6 m, the vertical accuracy of the tunnel was approximately 0.00–0.03 m, which is considered as relatively stable. The vertical accuracy of the overpass, intersection and freeway was also stable. Hence, the proposed method can be used to achieve stable and good accuracy in the vertical direction.

### 4.4. Discussion of the Main Factors Influence Registration Accuracy

Section 4.3.2 shows the registration accuracy evaluation results for each case area. In this subsection, the main influence factors are analyzed and discussed, including time synchronization error, GPS signal and vehicle speed.

#### 4.4.1. Time Synchronization for Different Sensors

The time system of an MMS is an important parameter as it determines the main accuracy of the MMS. Therefore, a vital step in data processing is time synchronization amongst the different sensors. Table 6 shows the time system used in the proposed MMS. The GPS, IMU and panoramic camera adopted GPS time, whereas the laser scanner adopted the time of the operating system (Windows time). GPS time is generally accurate, and Windows time is characterized by relatively low accuracy. Therefore, the error between the GPS time and Windows time will affect the registration results. During data collection, the synchronization error accumulates.

The time synchronization error will affect accuracy in the driving direction according to the following equation.

(13) ds=v×dt

where ds denotes the affect distance and dt denotes the time synchronization error. v is the vehicle speed. As per the preceding equation, registration accuracy is affected by the time synchronisation error. When the time synchronisation error was 1 ms (0.001 s) and the vehicle speed was 40 km/h, accuracy decreased by 1.1 cm. Therefore, prior to data collection, the system error between GPS time and Windows time needs to be calibrated; and after data collection, the same operation should also be conducted to improve the time accuracy of the laser scanner data.

#### 4.4.2. Vehicle Speed

As shown in Equation (13), the affect distance is associated with the time synchronization error as well as the speed of the vehicle. Vehicle speed was an important parameter when the MMS collected the data as it directly determined the efficiency of the MMS. The collection parameter of the other sensors was fixed during data collection, and only the speed of the vehicle was not determined. Therefore, the resolution of the point clouds will be affected by the vehicle’s speed and when compared with the dense point clouds, registration with sparse point clouds will obviously lead to lower accuracy. Therefore, for the data acquisition system concerned, the vehicle speed should be not as fast as possible.

Given that the vehicle speed was 30 km/h and the frequency of the laser scanner was 100 Hz, the resolution of the point clouds along the driving direction was 0.083 m. Once the speed increased, the resolution decreased rapidly and led to less registration accuracy.

#### 4.4.3. Positioning Error

**Figure 11:**
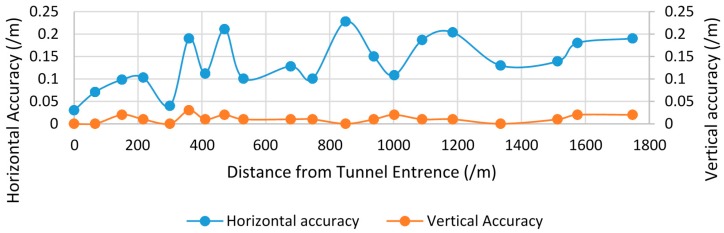
Spatial distribution of the check points’ accuracy in the tunnel case.

GPS was one of the main positioning sensors installed in the MMS. This sensor determined the location of the vehicle and provided it to the IMU to obtain accurate orientation parameters of the vehicle. Thus, a lack of GPS signal will affect MMS positioning and registration accuracy. Although vehicle position can be obtained by other sensors, including the angular sensor for wheels and IMU data, when the GPS signal is unlocked, the positioning error accumulates. In our studies, the GPS signal was unlocked only in the tunnel case. The spatial distribution of the checkpoints’ accuracy associated with mileage is shown in the Figure 11. The mileage can be regarded as the time after the vehicle entered the tunnel.

As seen in Figure 11, checkpoint accuracy decreased with mileage. Therefore, if a tunnel is too long, registration accuracy cannot be guaranteed. The vertical accuracy as relatively stable and varied from 0.00 to 0.02 m as the road area around the symbols was flat. Even when the horizontal difference is large, given that the road is flat, the vertical difference remains stable.

## 5. Discussion and Conclusions

An invisible feature point, which was calculated based on the sensor constellation, was hired for registration between the road’s point clouds with the panoramic imagery. The feature point, which is the intersection of the connecting line between the GPS antenna and the panoramic camera with a horizontal plane, was utilized to separate the large volume point clouds into blocks. This invisible feature point was fixed after the sensor constellation was determined and was non-relevance with the environment. Therefore, it can always be calculated during data processing. This ensures the 1:1 matching and thus increases the possibility of successful registration.

Four typical road types—overpass, freeway, tunnel, and surface roads—were selected to verify the proposed method. Our results show that most of the point clouds (with an average of 99.7%) successfully registered with the panoramic images with a high efficiency. Geometric evaluation results indicate that horizontal accuracy was approximately 0.10–0.20 m, and vertical accuracy was approximately 0.01–0.02 m for all cases.

The novelty of the proposed method is that an invisible feature point was calculated according to the sensor constellation, mainly the GPS and panoramic camera. As the constellation of the GPS and panoramic camera was fixed after the sensors were installed, and both sensors contained positioning modules, the feature point can always be found during travel. This improved the stability and reduced the complexity of registration computing.

In this paper, the segmentation feature point was computed based on the real-time position of the GPS and panoramic camera. However, if the sensor constellation is different to the MMS proposed in this paper, users may alter a different feature point to segment the point clouds. The selection of the segmentation feature should satisfy two conditions: first, the feature point should be fixed and easy to find after the sensors are installed; and second, the calculation of the feature point should be simple and with no iteration required. These will ensure the accuracy and efficiency of registration.

Although the calculation method in this study is relatively simple and uses only one uncertain parameter ($W_{back}$), this parameter is important for registration as it determines the width of the central blocks. An unmatched part will exist if too small a value is selected, and a mixture part will be available if a large value is used during the registration. We recommend a selection of $W_{back}$ according to the urban environment. For example, if the MMS operates in a freeway, then the $W_{back}$ parameter can be determined by the width of the lanes, including the emergency lane. This condition means that the first laser scanner, LS-1, will always be used to capture the point clouds in the pavement of the road.

Another important parameter in the proposed method is the road inclination angle β, which is computed during the registration for each moment when the panoramic camera operates. The road’s point clouds around the area are used to fit the inclination angle at that time and location. Furthermore, the adjacent epochs of GPS can be used to calculate the inclination angle. Therefore, the proposed method cannot be directly used in a real-time system; however, once the slopes of the roads are available for a city, the proposed method can be used to register the point clouds with panoramic images in real-time.

## Figures and Tables

**Table 1:** Image search range of each laser scanner.

Laser Scanner	Lens of Panoramic Camera
LS-1	Lens-0
LS-2	Lens-1, Lens-2, Lens-5
LS-3	Lens-3, Lens-4, Lens-5

**Table 2:** Environment and main parameters of data collection.

Case Type	Overpass	Freeway	Tunnel	Surface Roads
Environment complexity	Complex	Simple	Simple	Complex
GPS signal	Good	Good	None	Average
Length (km)	30.8	27.5	2.0	11.6
Average speed (km/h)	30	40	30	22
Time span (min)	61.50	41.27	3.34	31.75

**Table 3:** Efficiency evaluation of the different laser scanner’s point clouds in each dataset.

Type	Laser Scanner	Total Points	Matched Points	Match Rate (%)	Computation Time (s)
Overpass	LS-1	120,210,560	120,099,095	99.91	3163
	LS-2	46,040,486	45,750,431	99.37	1972
	LS-3	48,009,700	47,625,623	99.20	2060
Freeway	LS-1	72,612,193	72,601,383	99.98	1910
	LS-2	26,218,503	26,207,957	99.95	1092
	LS-3	28,495,605	28,486,135	99.96	1187
Surface roads	LS-1	61,756,177	61,695,284	99.90	1625
	LS-2	23,249,154	23,238,371	99.95	968
	LS-3	26,393,008	26,381,647	99.95	1099
Tunnel	LS-1	8,582,302	8,511,419	99.79	199
	LS-2	4,797,171	4,787,142	99.17	225
	LS-3	6,002,796	5,971,450	99.47	250

**Table 4:** Average computation efficiency evaluation.

Laser Scanner	Total Points	Matched Point	Total Computation Time (s)	Average Computation Efficiency
LS-1	263,161,232	262,907,161	6897	38,155
LS-2	101,305,314	99,983,901	4257	23,870
LS-3	108,901,109	108,464,855	4596	24,006

**Table 5:** Average geometric accuracy evaluation results (m).

Index	Case Area	$d_{H}$			$d_{V}$			d		
		Min.	Max.	Avg.	Min.	Max.	Avg.	Min.	Max.	Avg.
**1**	Overpass	0.000	0.340	0.178	0.000	0.030	0.009	0.000	0.352	0.179
**2**	Intersection	0.031	0.393	0.139	0.000	0.020	0.011	0.033	0.394	0.140
**3**	Tunnel	0.030	0.228	0.135	0.000	0.020	0.011	0.030	0.228	0.135
**4**	Freeway	0.000	0.160	0.079	0.000	0.010	0.020	0.000	0.161	0.112

**Table 6:** Time system used in the MMS.

Sensor	Time System
GPS	GPS time
IMU	GPS time
Panoramic camera	GPS time
Laser scanner	Windows time

## Appendix A

**Table A1:** Checkpoints of overpass case (m).

ID	Before Registration			After Registration			$d_{H}$	$d_{V}$	d
	X	Y	Z	X	Y	Z			
1	557,266.54	3,464,931.96	5.95	557,266.54	3,464,931.96	5.95	0.00	0.00	0.00
2	558,161.27	3,465,074.76	5.89	558,161.30	3,465,074.78	5.89	0.04	0.00	0.04
3	558,049.90	3,465,035.68	5.88	558,049.81	3,465,035.66	5.91	0.09	0.03	0.10
4	557,132.70	3,466,020.56	5.73	557,132.70	3,466,020.46	5.74	0.10	0.01	0.10
5	557,113.87	3,466,025.27	5.75	557,113.87	3,466,025.37	5.75	0.10	0.00	0.10
6	55,581.99	3,465,065.84	5.82	555,811.08	3,465,065.73	5.82	0.14	0.00	0.14
7	556,456.29	3,465,032.10	7.29	556,456.19	3,465,032.16	7.30	0.12	0.01	0.12
8	556,885.17	3,465,062.65	10.97	556,885.06	3,465,062.53	10.96	0.16	0.01	0.16
9	556,547.91	3,465,037.44	6.99	556,547.81	3,465,037.5	7.00	0.12	0.01	0.12
10	557,127.50	3,466,267.06	5.86	557,127.36	3,466,266.96	5.84	0.17	0.02	0.17
11	557,108.58	3,465,007.55	5.47	557,108.63	3,465,007.78	5.46	0.24	0.01	0.24
12	558,050.04	3,465,035.63	5.99	558,049.75	3,465,035.67	5.98	0.29	0.01	0.29
13	557,130.75	3,464,592.06	5.92	557,130.82	3,464,592.06	5.92	0.07	0.00	0.07
14	557,131.73	3,465,560.56	7.81	557,131.71	3,465,560.38	7.84	0.18	0.03	0.18
15	557,118.36	3,465,905.69	5.88	557,118.45	3,465,905.49	5.88	0.22	0.00	0.22
16	557,144.34	3,464,662.67	5.66	557,144.32	3,464,662.33	5.66	0.34	0.00	0.34
17	556,322.90	3,465,038.68	7.58	556,322.63	3,465,038.75	7.59	0.28	0.01	0.28
18	558,458.61	3,465,031.80	5.81	558,458.40	3,465,031.94	5.80	0.25	0.01	0.25
19	557,388.51	3,465,059.40	9.48	557,388.86	3,465,059.36	9.47	0.35	0.01	0.35
20	555,258.91	3,465,052.20	7.46	555,259.19	3,465,052.06	7.46	0.31	0.00	0.31

**Table A2:** Checkpoints of freeway case (m).

ID	Before Registration			After Registration			$d_{H}$	$d_{V}$	d
	X	Y	Z	X	Y	Z			
1	529,034.53	3,426,244.34	7.09	529,034.53	3,426,244.34	7.09	0.00	0.00	0.00
2	529,278.04	3,424,996.51	13.86	529,278.04	3,424,996.51	13.86	0.00	0.00	0.00
3	529,296.45	3,423,696.15	6.23	529,296.45	3,423,696.15	6.23	0.00	0.00	0.00
4	529,601.83	3,422,438.62	6.41	529,601.90	3,422,438.66	6.42	0.08	0.01	0.08
5	530,364.20	3,421,574.78	6.68	530,364.23	3,421,574.81	6.69	0.04	0.01	0.04
6	531,210.65	3,420,911.27	7.35	531,210.69	3,420,911.32	7.34	0.06	0.01	0.06
7	531,896.87	3,420,146.05	5.94	531,896.92	3,420,146.07	5.94	0.05	0.00	0.05
8	532,229.98	3,419,233.14	5.86	532,230.08	3,419,233.16	5.86	0.10	0.00	0.10
9	532,474.61	3,418,398.48	7.56	532,474.69	3,418,398.40	7.55	0.11	0.01	0.11
10	532,079.20	3,417,664.18	6.25	532,079.23	3,417,664.06	6.25	0.12	0.00	0.12
11	532,531.10	3,417,673.95	7.63	532,530.99	3,417,673.98	7.64	0.11	0.01	0.11
12	532,504.34	3,418,387.89	7.60	532,504.34	3,418,387.89	7.60	0.00	0.00	0.00
13	532,251.38	3,419,256.93	6.05	532,251.36	3,419,256.79	6.06	0.14	0.01	0.14
14	531,904.05	3,420,172.37	6.27	531,903.94	3,420,172.31	6.27	0.13	0.00	0.13
15	531,224.95	3,420,924.95	7.56	531,224.96	3,420,924.79	7.57	0.16	0.01	0.16
16	530,387.18	3,421,581.64	6.96	530,387.20	3,421,581.51	6.97	0.13	0.01	0.13
17	529,614.89	3,422,455.49	6.17	529,614.77	3,422,455.43	6.18	0.13	0.01	0.13
18	529,316.68	3,423,691.46	6.09	529,316.59	3,423,691.46	6.09	0.09	0.00	0.09
19	529,301.14	3,424,989.35	13.86	529,301.14	3,424,989.35	13.86	0.00	0.00	0.00
20	529,073.28	3,426,233.00	7.77	529,073.23	3,426,233.09	7.77	0.10	0.00	0.10

**Table A3:** Checkpoints of tunnel case (m).

ID	Before Registration			After Registration			$d_{H}$	$d_{V}$	d
	X	Y	Z	X	Y	Z			
1	554,018.04	3,464,962.70	−5.59	554,018.04	3,464,962.67	−5.59	0.03	0.00	0.03
2	553,953.54	3,464,949.68	−8.90	553,953.55	3,464,949.61	−8.90	0.07	0.00	0.07
3	553,871.04	3,464,933.35	−13.28	553,870.95	3,464,933.31	−13.26	0.10	0.02	0.10
4	553,805.49	3,464,922.17	−16.53	553,805.40	3,464,922.12	−16.52	0.10	0.01	0.10
5	553,722.65	3,464,911.07	−20.63	553,722.65	3,464,911.11	−20.63	0.04	0.00	0.04
6	553,663.13	3,464,905.16	−23.48	553,663.32	3,464,905.15	−23.45	0.19	0.03	0.19
7	553,612.16	3,464,901.45	−25.88	553,612.26	3,464,901.40	−25.87	0.11	0.01	0.11
8	553,551.50	3,464,898.54	−28.75	553,551.71	3,464,898.52	−28.73	0.21	0.02	0.21
9	553,491.84	3,464,897.35	−31.51	553,491.74	3,464,897.36	−31.50	0.10	0.01	0.10
10	553,342.61	3,464,901.97	−38.04	553,342.71	3,464,901.89	−38.03	0.13	0.01	0.13
11	553,273.86	3,464,907.56	−40.30	553,273.76	3,464,907.57	−40.31	0.10	0.01	0.10
12	553,169.33	3,464,920.51	−40.67	553,169.19	3,464,920.33	−40.67	0.23	0.00	0.23
13	553,080.47	3,464,935.61	−38.84	553,080.35	3,464,935.52	−38.85	0.15	0.01	0.15
14	553,016.10	3,464,948.96	−37.44	553,016.16	3,464,948.87	−37.42	0.11	0.02	0.11
15	552,928.97	3,464,970.59	−35.29	552,928.79	3,464,970.54	−35.28	0.19	0.01	0.19
16	552,833.60	3,464,998.49	−31.51	552,833.80	3,464,998.53	−31.50	0.20	0.01	0.20
17	552,686.29	3,465,051.83	−24.51	552,686.17	3,465,051.78	−24.51	0.13	0.00	0.13
18	552,514.30	3,465,128.75	−15.75	552,514.17	3,465,128.70	−15.76	0.14	0.01	0.14
19	552,454.70	3,465,156.64	−12.71	552,454.52	3,465,156.65	−12.73	0.18	0.02	0.18
20	552,290.84	3,465,229.46	−4.36	552,290.65	3,465,229.47	−4.38	0.19	0.02	0.19

**Table A4:** Checkpoints of surface roads case (m).

ID	Before Registration			After Registration			$d_{H}$	$d_{V}$	d
	X	Y	Z	X	Y	Z			
1	551,179.32	3,451,668.88	5.57	551,179.28	3,451,668.87	5.57	0.04	0.00	0.04
2	551,244.46	3,451,317.43	5.56	551,244.43	3,451,317.42	5.57	0.03	0.01	0.03
3	551,341.51	3,451,259.73	6.26	551,341.46	3,451,259.65	6.26	0.09	0.00	0.09
4	551,487.13	3,450,511.96	7.77	551,487.12	3,450,512.06	7.79	0.10	0.02	0.10
5	551,361.83	3,450,949.48	7.66	551,361.84	3,450,949.40	7.68	0.08	0.02	0.08
6	551,013.69	3,451,205.67	5.76	551,013.70	3,451,205.64	5.75	0.03	0.01	0.03
7	551,253.57	3,451,315.07	5.58	551,253.54	3,451,315.06	5.59	0.03	0.01	0.03
8	551,165.41	3,451,722.61	5.81	551,165.39	3,451,722.44	5.83	0.17	0.02	0.17
9	551,458.43	3,450,603.59	10.52	551,458.47	3,450,603.60	10.52	0.04	0.00	0.04
10	551,532.58	3,450,377.08	6.07	551,532.62	3,450,377.11	6.06	0.05	0.01	0.05
11	551,308.99	3,451,118.60	5.32	551,308.93	3,451,118.58	5.32	0.06	0.00	0.06
12	551,197.67	3,451,603.26	5.49	551,197.76	3,451,603.20	5.47	0.11	0.02	0.11
13	551,114.85	3,451,986.20	5.57	551,114.90	3,451,986.13	5.58	0.09	0.01	0.09
14	551,043.35	3,452,331.86	5.55	551,043.40	3,452,331.59	5.55	0.27	0.00	0.27
15	550,908.49	3,452,987.78	7.50	550,908.52	3,452,987.57	7.50	0.21	0.00	0.21
16	550,873.00	3,453,263.91	12.26	550,872.98	3,453,264.28	12.27	0.37	0.01	0.37
17	551,009.54	3,452,439.48	5.86	551,009.49	3,452,439.87	5.89	0.39	0.03	0.39
18	551,132.00	3,451,866.43	5.82	551,132.06	3,451,866.35	5.85	0.10	0.03	0.10
19	551,101.99	3,452,047.10	5.51	551,102.07	3,452,046.83	5.52	0.28	0.01	0.28
20	550,984.51	3,452,623.22	6.30	550,984.55	3,452,623.00	6.30	0.22	0.00	0.22
